# Cgrp Characterization And Classification

**DOI:** 10.1100/tsw.2001.427

**Published:** 2001-11-09

**Authors:** Ian Marshall

**Affiliations:** Department of Pharmacology, University College London, Gower Street, London, WC1E 6BT, UK

## Abstract

The peptide CGRP is thought to be an agonist at a number of receptors including its own as well as those for adrenomedullin and amylin. CGRP is thought to act through its own receptors if it is antagonized by the truncated peptide CGRP8-37 although this assumes the specificity of the antagonist.

